# Reproductive health of male Australian veterans of the 1991 Gulf War

**DOI:** 10.1186/1471-2458-7-79

**Published:** 2007-05-16

**Authors:** Helen L Kelsall, Malcolm R Sim, Jillian F Ikin, Andrew B Forbes, Dean P McKenzie, Deborah C Glass, Peter Ittak

**Affiliations:** 1 Department of Epidemiology and Preventive Medicine, Monash University – Central and Eastern Clinical School, Alfred Hospital, Commercial Rd, Melbourne, Victoria 3004, Australia

## Abstract

**Background:**

Since the 1991 Gulf War concerns have been raised about the effects of deployment to the Gulf War on veterans' health. Studies of the reproductive health of Gulf War veterans have reported varied findings.

**Methods:**

We undertook a cross-sectional study of male Australian Gulf War veterans (n = 1,424) and a randomly sampled military comparison group (n = 1,548). The study was conducted from August 2000 to April 2002. A postal questionnaire included questions about difficulties achieving pregnancy, pregnancy outcomes including live births, stillbirths, miscarriages and terminations; and for all live births gestation, birth weight, sex, and any cancers, birth defects, chromosomal abnormalities or serious health problems.

**Results:**

Male Gulf War veterans reported slightly increased risk of fertility difficulties following the Gulf War (odds ratio [OR] 1.4; 95% confidence interval [CI] 1.0–1.8), but were more successful at subsequently fathering a child (OR 1.8; 95% CI 1.3–2.6). The study groups reported similar rates of pregnancies and live births. There was no increased risk in veterans of miscarriage, stillbirth, or terminations. Children of male Gulf War veterans born after the period of the Gulf War were not at greater risk of being born prematurely, having a low birth weight, or having a birth defect or chromosomal abnormality (OR 1.0; 95% CI 0.6–1.6). The numbers of cancers and deaths in children were too small to draw any firm conclusions.

**Conclusion:**

The results of this study do not show an increased risk of adverse reproductive outcome in Australian male Gulf War veterans.

## Background

Since the 1990–91 Gulf War, concerns have been raised about possible deployment-related effects upon the reproductive health of Gulf War veterans (veterans). The findings of studies investigating reproductive health and the risk of reported birth defects [1-4] and specific types of birth defects [3,5-8] in offspring of veterans from several countries have been varied. Some adverse reproductive outcomes have been reported more commonly by US, UK and Canadian veterans compared with non-Gulf comparison groups, including miscarriages [1-3] and birth defects [1-3,7]. Danish veterans and controls were similar with respect to sex hormone levels, fertility rates, number of children with congenital diseases including malformations, and abortion rates [9].

Birth defects among children born to veterans from two Mississippi National Guard Units [10], Canadian veterans [1] and US veterans [6] were comparable to those in the relevant general populations. No increase in the overall risk of birth defects in the offspring of Gulf War veterans was found in a retrospective study of live births in US military hospitals [6], nor in a study that ascertained births outside military hospitals and which investigated defects developing in the first year of life [5,8]. There is some evidence for increased risk of specific types of birth defects in the children of US veterans: tricuspid valve insufficiency, renal agenesis and hypoplasia in one birth defect registry based study [5] but not another [6]. Increased risk of malformations of the genitourinary and musculoskeletal systems, 'other' defects of the digestive system, and 'other' non-chromosomal (non-syndrome) malformations found in offspring of UK veterans [3] weakened when analyses were restricted to clinically confirmed conditions only. The small numbers of cases and wide confidence intervals indicated that the three-fold increased risk of Goldenhar Syndrome in infants born to US Gulf War veterans compared with non-deployed military personnel [7] should be interpreted with caution.

The United States General Accounting Office identified several substances that personnel could have been exposed to during the Gulf War that may cause reproductive dysfunction, including pesticides, substances found in the oil well fires and decontaminating agents [11,12]. However, the small numbers of individual birth defects have limited the ability of previous studies investigating associations with these types of Gulf War related exposures.

The aim of our study was to investigate whether male Australian Gulf War veterans had increased adverse reproductive outcomes (fertility difficulties, miscarriage, stillbirth or termination of pregnancies) or had increased adverse health outcomes (premature birth, low birth weight, cancers, birth defects or chromosomal abnormalities or death) in their live born children compared with a military comparison group following the period of the Gulf War.

## Methods

The study was approved by the Standing Committee on Ethics in Research Involving Humans at Monash University, the Australian Government Department of Veterans' Affairs Human Research Ethics Committee and the Australian Defence Human Research Ethics Committee.

### Study population

The study population was the entire cohort of 1,871 Australian veterans who served in the Gulf region during the period 2 August, 1990 to 4 September, 1991. The majority (84.4%) were naval personnel. A comparison group of 2,924 subjects was randomly selected from 26,411 Australian Defence Force personnel operational at the time of the Gulf War but not deployed to that conflict. The comparison group was frequency matched to the veteran group by sex, service type, and three-year age bands. The study was conducted from August 2000 to April 2002. Subjects were recruited via mailed invitation with two further mailings and follow-up phone contact for non-responders. Further details are provided by Ikin *et al *[13]. Overall, 80.5% of eligible veterans and 56.8% of eligible comparison group members participated. Due to the small numbers of female Gulf War veterans, the analyses of reproductive outcomes were limited to males.

The study groups for this analysis consisted of 1,424 male veterans and 1,548 male comparison group subjects who completed a postal questionnaire. Participating veterans were slightly younger, more likely to have served in the Navy, less highly ranked and less likely to have tertiary education, than comparison group participants. Marital status was similar for both groups. Recruitment, demographic characteristics, physical and psychological health status of participants, and the exposures reported by participants, have previously been reported [13-19].

### Data collection

Participants completed a self-administered postal questionnaire. Questions relating to fertility were based on those used in a previous Australian fertility study [20]. For male participants, fertility difficulties were defined as difficulties for the participant and their partner getting pregnant despite trying for at least 12 months. Participants were asked about the year that these difficulties began, whether they had sought or undertaken infertility treatment and, if so, whether a cause for their fertility difficulties had been found, and whether they had since fathered a pregnancy.

For all fathered pregnancies (irrespective of fertility status), participants were asked to report whether the pregnancy resulted in a live birth, miscarriage, stillbirth or termination and to provide the date for each of these outcomes. In relation to live births, participants were asked to report birth date, sex, birth weight, gestation, and whether any live born child had a birth defect or chromosomal abnormality, other serious health problem, developed cancer or had died.

Reported birth defects and chromosomal abnormalities were reviewed by medically qualified study team members (HK, MS) and excluded if they were clearly misclassified. Reports of other serious health problems that were identified after review as probable birth defects, according to established criteria [21], were included. Reported childhood cancers were matched against national cancer registry data.

Birth weights were categorised as 'low' (<2500 grams) or 'very low' (<1500 grams). Births were categorised as 'premature' if gestation was reported to be 36 weeks or less. Post-Gulf War fertility difficulties and adverse pregnancy outcomes were defined as those occurring in 1991 or later. The health of live born children is reported for children born in 1992 or later.

### Statistical analysis

Statistical analyses were performed using Stata 7.0 [22]. Associations between Gulf War deployment and fertility difficulties, adjusting for potential confounding factors, were assessed using logistic regression and reported as adjusted odds ratios (OR) with 95% confidence intervals (95% CI). Associations between Gulf War deployment and pregnancy outcomes occurring in 1991 or later, adjusting for potential confounding factors, were obtained using polytomous logistic regression [23]. Where a cell size was small (arbitrarily but conventionally defined as ≤5), exact logistic regression [24] was performed [25]. Standard errors and 95% Cis calculated in relation to pregnancy outcomes in 1991 or later, and for the health of live born children in 1992 or later, were adjusted for clustering of multiple pregnancies, or multiple live-births, within individuals respectively [26]. The values of the unadjusted and adjusted odds ratios were highly similar, and so only the adjusted results are reported.

## Results

### Fertility difficulties

Approximately 14% of veterans and 13% of the comparison group reported fertility difficulties in their lifetime (Table 1). Veterans were no more likely than the comparison group to have reported experiencing fertility difficulties prior to 1991, but more likely to have reported these occurring for the first time in 1991 or later. Of those subjects in both groups who reported fertility difficulties in 1991 or later, approximately half had sought infertility treatment and, of these, more than half reported that a cause for their fertility difficulties had been found. Veterans who reported fertility difficulties in 1991 or later were more likely than similar comparison group subjects to report that they had since fathered a child.

**Table 1:** Self-reported fertility difficulties in male Gulf War veteran and comparison group subjects

	**Gulf War veterans**		**Comparison group**			
	**n**	**(%)**	**n**	**(%)**	**Adj OR**^b^	**(95% CI)**

	**N = 1378**		**N = 1504**			

**Experienced difficulty getting pregnant pre-1991**	65	(4.7)	92	(6.1)		

	**N = 1313**^a^		**N = 1412**^a^			

**First experienced difficulty getting pregnant in 1991 or later**	130	(9.9)	102	(7.2)	1.4	(1.0–1.8)
Sought infertility treatment	53	(4.0)	47	(3.3)	1.2	(0.8–1.8)
Cause for infertility found	31	(2.4)	25	(1.8)	1.2	(0.7–2.1)
Fathered a pregnancy since then	85	(6.5)	52	(3.7)	1.8	(1.3–2.6)

^a ^Persons reporting fertility difficulties prior to 1991 are excluded.

^b ^Odds ratios were obtained by logistic regression adjusting for age, rank, service type, education, marital status, smoking and alcohol.

### Pregnancy outcomes

Live births, miscarriages, stillbirths and terminations reported to have occurred in 1991 or later are shown in Table 2. The pattern of these pregnancy outcomes in the study groups is very similar. Live births were the outcome for more than 80% of pregnancies in each group. The 1170 and 1272 live births were reported by 684 veterans (48%) and 732 comparison group subjects (47%) respectively (data not shown). These two groups both reported an average of 1.7 live births.

**Table 2:** Self-reported pregnancy outcomes in 1991 or later and reported birthweights and health outcomes for children born in 1992 or later

	**Gulf War veterans**		**Comparison group**			
**Pregnancy outcomes in 1991 or later**	**n**	**(%)**^a^	**n**	**(%)**^a^	**Adj OR**^b^	**95% CI**^c^

Number of pregnancies	N = 1448		N = 1555			
Live births	1170	(80.8)	1272	(81.8)	-	-
Miscarriages	204	(14.1)	197	(12.7)	1.1	(0.8–1.3)
Stillbirths	5	(0.4)	14	(0.9)		
Terminations	69	(4.8)	72	(4.6)	1.0	(0.7–1.5)

**Children born in 1992 or later**	**n**	**(%)**	**n**	**(%)**	**Adj OR**	**95% CI**^f^

Number of children	N = 1096	-	N = 1145	-		
Low birth weight	56	(6.3)^d^	65	(6.9)^d^	0.9	(0.6–1.3)
Very low birth weight	9	(1.0)^d^	9	(1.0)^d^	1.0	(0.4–2.8)
Premature birth	71	(7.3)^e^	94	(9.4)^e^	0.7	(0.5–1.1)
Reported cancer	4	(0.4)	1	(0.1)	4.2^g^	(0.5–38.0)^g^
Reported birth defect	40	(3.6)	38	(3.3)	1.0	(0.6–1.6)
Reported death	2	(0.2)	4	(0.3)	0.5^g^	(0.1–2.9)^g^

^a ^Percentage values for pregnancy outcomes in 1991 or later are derived from N = 1448 pregnancies in Gulf War veterans and N = 1555 pregnancies in the comparison group.

^b ^Odds ratios are for miscarriages/stillbirths combined, or terminations, compared to live births. Odds ratios were obtained by polytomous logistic regression adjusting for age, rank, service type, education, marital status, smoking and alcohol.

^c ^CIs are adjusted for clustering of multiple pregnancies within individuals.

^d ^These percentages are derived from N = 885 live births for Gulf War veterans and N = 936 live births for the comparison group for whom birth weight was provided.

^e ^These percentages are derived from N = 978 live births for Gulf War veterans and N = 997 live births for the comparison group for whom duration of pregnancy was provided.

^f ^Standard errors were adjusted for clustering of multiple births within the same individual.

^g ^Due to the small numbers of events, the odds ratio was not adjusted for confounders and clustering. The associated 95% CI was adjusted for clustering.

### Health of live born children

Reported birth weights and health outcomes for children born in 1992 or later are shown in Table 2. The 1,096 and 1,145 live births were reported by 665 veterans (47%) and 687 comparison group subjects (44%). The children of both study groups had a very similar pattern of birth weight, duration of gestation, and birth defects. The quality of the self-reported birth defect data was not high enough to enable further categorisation into major or minor congenital defects. The total numbers of childhood cancers and deaths were very small in both groups.

Of the four children of veterans who were born in 1992 or later and reported to have cancer, three were confirmed as having cancer by matching with the national cancer registry. The one comparison group child born in 1992 or later and reported to have cancer was not confirmed.

## Discussion

We found increased reporting of fertility difficulties following the time of the Gulf War in male Australian Gulf War veterans compared with a randomly sampled military comparison group, but veterans also reported greater success in subsequently fathering a child. Veterans and comparison group subjects reported similar rates of pregnancies and live births in the period since the Gulf War, and we found no evidence for an increased risk of stillbirths, miscarriages or terminations in veterans or an increased risk of birth defects in their live born children.

Our finding of an increase in reported fertility difficulties since the Gulf War is consistent with increased risk of failure to achieve conception or live births in UK veterans [27]. The likelihood of Australian or Danish [9] veterans seeking infertility treatment was not significantly different to their non-Gulf comparison groups. Using a similar, though stricter, definition for fertility difficulties, a study of Western Australian couples reported lifetime 'infertility' prevalence to be approximately 19% [28]; 5%–6% higher than that reported in 1991 or later by our study groups.

Our finding of similar reporting of miscarriages by veterans and comparison group subjects contrasts with the higher rates of reported miscarriages in male US and UK veterans [2,3], but is consistent with findings in a further study of US veterans [4] and in veterans from Denmark [9], another country with a comparatively small Gulf War contingent. Similar reporting of stillbirths between study groups is consistent across several studies [2-4,9].

We found no evidence for an increased risk of reported birth defects in male Australian veterans, in contrast with the increased self-reporting of birth defects in Canadian veterans [1], who were also mainly naval personnel, and in male UK [3] and US [2] veterans. Our study's findings were consistent with findings of no increased birth defect risk overall in Danish veterans [9], in live births to US veterans in military hospitals [6], and when births outside military hospitals and defects developing in the first year of life were also ascertained [5]. Australian national data estimates the rates of birth defects or congenital malformations from 1981–96 to be 160.1 per 10,000 live births (1.6%) [21], a rate half that reported by our study groups. However this national data set only included major birth defects.

Several limitations to our study need to be acknowledged. The numbers of adverse reproductive outcomes reported by the study groups for the period following the Gulf War were quite small, limiting the power of the study to identify small differences in risk. Review of reported birth defects to improve classification was to some extent limited by the quality of the reports. By limiting the reporting of birth defects to those in live born children, cases of severe birth defects may have been excluded. The small number of birth defects in our study groups did not allow categorisation into birth defect types. The numbers of reported cancers and deaths in children were too few in both study groups to be meaningfully interpreted. We also had insufficient numbers to investigate adverse reproductive outcomes in female veterans. Our study groups were categorised as exposed or unexposed on the basis of their deployment or not to the 1990–91 Gulf War. The lack of reliable exposure data on specific exposures in theatre and other occupational and behavioural characteristics that may adversely affect reproductive health is a further limitation of our study.

Adverse reproductive health outcomes were self-reported, and although we sought registry confirmation of reported cancers and deaths for children, our study was not able to seek medical confirmation of reported birth defects. In Australia there is no national birth defects register that contains identifying details that can be used for record linkage with studies such as ours, and the existing state and territory registers have not employed a standardised approach to data collection or coding.

Notwithstanding the limitations, our study has several advantages. We investigated the entire cohort of Australian Gulf War veterans, achieved a high response rate (80.5%), and compared veterans with a randomly sampled military comparison group. Despite a rigorous contact and recruitment strategy, the comparison group participation rate was lower than that of the veteran group, but was comparable to or greater than that of other major postal surveys of veterans [2,29,30]. Our formal evaluation of possible participation bias previously reported in relation to symptoms and medical conditions [14,15], psychological [13] and neurological [31] health suggests that this is unlikely to fully explain the differences (or lack thereof) that we found between our study groups.

## Conclusion

Australian male Gulf War veterans and a military comparison group had similar rates of pregnancies and live births in the period since the Gulf War and there appear to be no differences in the risk of stillbirths, miscarriages or terminations and no differences in the risk of birth defects. Although we found increased reporting of fertility difficulties following the time of the Gulf War in male veterans, this did not seem to be a long-lasting effect as veterans also reported greater success in subsequently fathering a child. Our findings are limited by small numbers of adverse reproductive outcomes, the reliance on self-reported data, and the difficulties in attempting to validate the data with medical records and birth defects registries. This study group warrants further investigation in time should a more accessible central national birth defects register be established.

## Competing interests

The author(s) declare that they have no competing interests.

## Authors' contributions

All authors participated in the design of the study and interpretation of the data. AF and DM performed the statistical analyses. HK drafted and revised the manuscript. Authors provided comments on the manuscript and read and approved the final manuscript.

## Pre-publication history

The pre-publication history for this paper can be accessed here:


